# The Synthesis, Metal Exchange, and Hyaluronate Functionalization of a Cationic Gallium-Based Thiosemicarbazone Anticancer Drug

**DOI:** 10.3390/molecules31030577

**Published:** 2026-02-06

**Authors:** Ye Ning, Meng-Lin Dong, Wen-Hua Zhang, David J. Young

**Affiliations:** 1 College of Chemistry, Chemical Engineering, and Materials Science, Soochow University, Suzhou 215123, China; 2 James Watt School of Engineering, Glasgow University, University Avenue, Glasgow G12 8QQ, UK

**Keywords:** gallium-based drugs, thiosemicarbazone, anticancer drugs, drug delivery, metal exchange

## Abstract

We herein demonstrate that the thiosemicarbazone (TSC) ligand N′-(di(pyridin-2-yl)methylene)-4-(thiazol-2-yl)piperazine-1-carbothiohydrazide (H**L**) can coordinate with Ga^3+^ to give cationic complex **[Ga(L)_2_]NO_3_** featuring an octahedral Ga(III) center. **[Ga(L)_2_]NO_3_** undergoes metathesis with both Fe^2+^ and Fe^3+^, resulting in the formation of respective Fe^2+^- and Fe^3+^ complexes. **[Ga(L)_2_]NO_3_** is also susceptible to anion exchange with sodium hyaluronate (NaA) to produce the nanoformulation **[Ga(L)_2_]A** with boosted aqueous solubility and cell targeting. **[Ga(L)_2_]A** demonstrated remarkable in vitro cytotoxicity against NCI-H82 and A549 (lung cancer), as well as KYSE-510 and Te-1 (esophageal cancer) cell lines, featuring half maximal inhibitory concentration (IC_50_) values in the range 0.102–2.616 μmol L^−1^. This work highlights the potential of using non-toxic and biocompatible Ga^3+^ as the central ion to prepare TSC-based nanomedicines for combating cancer.

## 1. Introduction

Thiosemicarbazones (TSCs) are a class of Schiff base that have been widely documented as highly promising anticancer drugs for a broad spectrum of cancer types through diverse mechanisms [1,2,3,4]. As N- and S-rich metal chelators, TSCs can elicit their anticancer effect by quick sequestration of cell-proliferative-dependent Fe^3+^ ions [4]. The anticancer effect of TSCs is profoundly affected by their structures [2,4,5,6]. In particular, the presence of an additional N coordination site, such as an α-pyridyl moiety, enables the formation of a more stable N, N, S tridentate chelate for more effective ion removal [7,8,9]. The promising anticancer potential of TSCs has been showcased by several drugs that have successfully entered clinical trials, such as Triapine, 5-HP, and COTI-2 [10,11,12,13].

Gallium (Ga), though with limited physiological function in the human body, has demonstrated important biological properties [14,15]. For example, Ga^3+^ is effective in suppressing bone resorption and simultaneously elevating plasma calcium [15]. It is used clinically for the treatment of hypercalcemia of malignancy and Paget’s disease of the bones [16]. Gallium(III) has also shown clinical efficacy in suppressing osteolysis and bone pain associated with multiple myeloma and bone metastases, and has been suggested as a treatment for osteoporosis [14].

It is notable that Ga^3+^ and Fe^3+^ share very similar charge-to-radius ratios, and that the chemical behavior of Ga^3+^ closely resembles that of Fe^3+^ [15,17,18]. Consequently, Ga^3+^ can bind to the iron-transport protein transferrin (Tf), although the affinity of Tf for Fe^3+^ is much higher than that for Ga^3+^, and the replacement of Ga^3+^ by Fe^3+^ occurs very slowly [15]. By comparison, Ga^3+^ is unable to enter Fe^2+^-containing proteins, such as hemoglobin and cytochromes. Hence, it does not enter red blood cells and does not interfere with oxygen transport [15].

Intrigued by this similarity between Ga^3+^ and Fe^3+^, an ion that is so critical to the metabolism of cancer cells, Ga(III)-based drugs have been widely developed to target Fe^3+^-dependent metabolism [6,19,20,21]. For example, tris(8-quinolinolato)-gallium(III) (KP46), originally demonstrated to show better bioavailability and higher toxicity than GaCl_3_ upon oral administration, is now used for treating various cancers, such as melanoma and renal cell carcinoma [19]. KP46 is also found to sensitize resistant leukemia cells and overcome Bcl-2-induced multidrug resistance in lymphoma cells via up-regulation of Harakiri and down-regulation of XIAP in vitro [20]. Ga(III) complexes with cloxyquin ligands have also been reported to induce ferroptosis in cancer cells [21]. Using human cancer cell lines 41M and SK-BR-3 as models, Arion and Keppler et al. revealed that the coordination of Ga^3+^ to TSCs unequivocally and specifically modulated cytotoxic potency in a beneficial manner, whereas surrogation of Ga^3+^ with Fe^3+^ impaired biological activity [6].

In our recent work, we reported the synthesis of a new TSC-based anticancer drug of N′-(di(pyridin-2-yl)methylene)-4-(thiazol-2-yl)piperazine-1-carbothiohydrazide (H**L**) featuring the di-2-pyridylketone moiety with chelating-bridging functions [22]. Ligand H**L** can readily associate with Cu^2+^ to give the corresponding coordination complexes that demonstrated superior anticancer performances against hepatocellular carcinoma by reactive oxygen species generation. Pharmacokinetic studies also revealed that H**L** can be successfully absorbed via oral administration, with a favorable half-life that was nearly double that of intravenous administration, rendering H**L** a clinically promising oral drug.

**Scheme 1:**
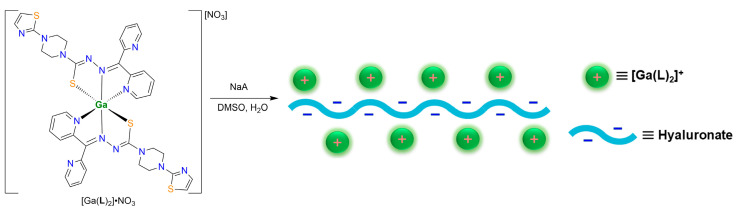
Synthesis of **[Ga(L)_2_]NO_3_** and its anion exchange with NaA to give **[Ga(L)_2_]A**.

**Figure 1:**
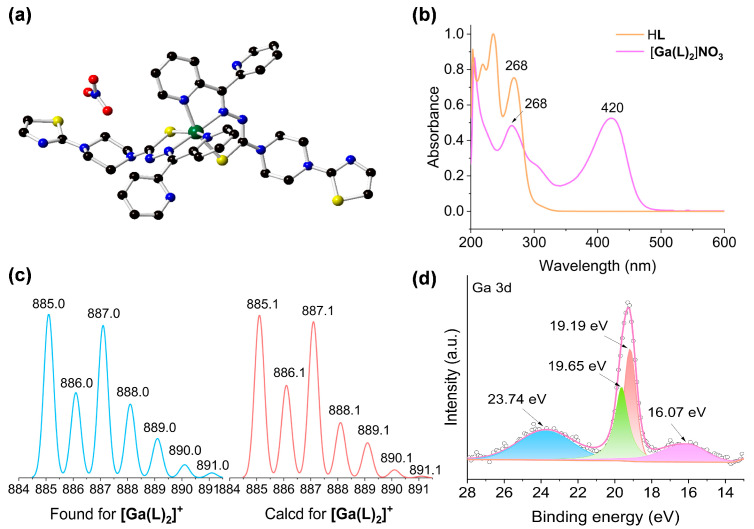
The crystal structure of **[Ga(L)_2_]NO_3_** (**a**). The UV-Vis spectra of H**L** and **[Ga(L)_2_]NO_3_** in CH_2_Cl_2_ solution (**b**). The HPLC-MS results of **[Ga(L)_2_]NO_3_**, as demonstrated by the consistency between the experimental (blue) and calculated (orange) peak values and patterns (**c**) assignable as **[Ga(L)_2_]^+^**. The Ga 3d XPS spectra of **[Ga(L)_2_]NO_3_** (**d**). For (**a**), the MeOH solvate and hydrogen atoms are omitted for clarity. Color legend: Ga (dark green), S (yellow), O (red), N (blue), and C (black).

Herein, we explore the reaction of H**L** with Ga(NO_3_)_3_·9H_2_O and obtain the anticipated octahedral complex **[Ga(L)_2_]NO_3_** (Scheme 1 and Figure 1a). **[Ga(L)_2_]NO_3_** underwent metathesis with both Fe^2+^ and Fe^3+^ to yield the respective Fe^2+^- and Fe^3+^-based complexes quantitatively. The ionic nature of **[Ga(L)_2_]NO_3_** further permitted its anion exchange reaction with sodium hyaluronate (NaA) to give the corresponding nanoformulation **[Ga(L)_2_]A** to endow the material with aqueous solubility and targetability (Scheme 1) [23,24,25]. Our preliminary results suggest that **[Ga(L)_2_]A** exhibits pronounced cytotoxicity in cell lines NCI-H82 and A549 (lung cancer), as well as KYSE-510 and Te-1 (esophageal cancer). This work highlights the potential of anticancer nanomedicines from non-toxic and biocompatible Ga^3+^ and TSC ligands.

## 2. Results and Discussion

### 2.1. Synthesis and Structure of [Ga(L)_2_]NO_3_

The ligand H**L** was synthesized according to our previously reported procedures [22]. H**L** and similar ligand types featuring N, N, S-chelation functions are demonstrated to associate with diverse metal ions, particularly the biologically relevant Fe^2+/3+^ [6,26,27], Cu^+/2+^ [28,29,30], and Zn^2+^ [31,32], due to their soft-hard hybrid combinations. The reaction of Ga(NO_3_)_3_·9H_2_O and H**L** in MeOH under ambient conditions, followed by ether diffusion, gave rise to **[Ga(L)_2_]NO_3_** as yellow block crystals in 23.3% yield. Crystals of **[Ga(L)_2_]NO_3_** were soluble in common solvents ranging from polar to medium-polar solvents, such as H_2_O, MeOH, DMSO, DMF, and CH_2_Cl_2_.

Compound **[Ga(L)_2_]NO_3_** crystallized in the monoclinic space group *P*2_1_*/n* (Table 1), and its asymmetric unit contained a full **[Ga(L)_2_]NO_3_** molecule along with a MeOH solvate (Figure 1a). The octahedral coordination preference of Ga^3+^ enabled the chelation of a pair of ligands to give the [Ga(**L**)_2_]^+^ cation, in which a pair of N_py_ and a pair of S atoms are in *cis* positions, respectively. This is in addition to the *trans* arrangement of the N_hydrazone_ pair. The use of a tridentate ligand such as H**L** for octahedral complex precludes the formation of possible and unwanted ∆/Λ enantiomers typically found for bidentate ligands, such as dipyridyls and phenylpyridines [33]. The [Ga(**L**)_2_]^+^ cation was accompanied by an NO_3_^−^ counterion, further indicating that the ligand is tautomerized toward its enol form (thiol), which is then subsequently deprotonated [34,35].

In **[Ga(L)_2_]NO_3_**, the Ga–S bond distances (2.3600(6) and 2.3715(6) Å, Table 2) were similar to those reported in the literature, such as [bis(acetylpyrazine N, N-dimethylthiosemicarbazonato)-N, N, S-gallium (III)] hexafluorophosphate (2.3321(7) and 2.3532(9) Å) [6], [bis(acetylpyrazine N-pyrrolidinylthiosemicarbazonato)-N, N, S-gallium(III)] hexafluorophosphate (2.3389(6) and 2.3525(6) Å) [6]. It should be noted that, although Ga^3+^ and Fe^3+^ share a similar ionic radius, their respective Fe^3+^-based complexes using the same TSC ligand exhibit marginally shorter Fe–S bond distances. For example, the Fe–S bond distances in [bis(acetylpyrazine N, N-dimethylthiosemicarbazonato)-N, N, S-iron(III)] tetrachloroferrate(III) are 2.2242(6) and 2.2341(7) Å [6], similar to those found in [bis(acetylpyrazine N-pyrrolidinylthiosemicarbazonato)-N, N, S-iron(III)] tetrachloroferrate(III) (2.2156(9) and 2.2322(10) Å) [6]. A similar trend is observed in other Fe-TSC complexes, such as the Fe^3+^ complex 4-(4-nitrophenyl)-1-((pyridin-2-yl)methylene)thiosemicarbazide (2.2161(6) and 2.2009(9) Å) [36]. The shorter, more stable Fe–S bond distances compared to those of Ga–S probably serve as the driving force for their metathesis.

Platon void calculation also suggested that the guest-accessible void of **[Ga(L)_2_]NO_3_** occupies 7.4% (325.7 Å^3^ of 4381.8 Å^3^) of the total cell volume, and these voids are located with MeOH solvates (Figure S1) that are hydrogen-bonded to the free NO_3_^−^ (Table S1) [37].

### 2.2. Spectroscopic and Spectrometric Characterization of [Ga(L)_2_]NO_3_

High-performance liquid chromatography–mass spectrometry (HPLC-MS) analysis revealed signals at 885.0 m/z for **[Ga(L)_2_]NO_3_**, corresponding to **[Ga(L)_2_]^+^** (calculated 885.1 m/z) (Figure 1c). The ^1^H nuclear magnetic resonance (^1^H NMR) spectra showed that the thiosemicarbazone proton peak at 14.64 ppm in H**L** is absent in **[Ga(L)_2_]NO_3_** (Figure S2), providing evidence of the thione-to-thiol tautomerism and subsequent Ga^3+^ coordination by eliminating the thiol proton.

Fourier-transform infrared (FT-IR) spectroscopic analysis revealed significant coordination-induced modifications in the vibrational modes. The ν_(C=S)_ vibration at 869 cm^−1^ in the free ligand H**L** bathochromically shifted to 854 cm^−1^ in **[Ga(L)_2_]NO_3_** (Figure S3) [38]. By contrast, the C=N stretching vibration of H**L**, originally observed at 1579 cm^−1^ in the FT-IR spectrum, hypsochromically shifted to 1598 cm^−1^ upon coordination with Ga^3+^ in **[Ga(L)_2_]NO_3_** [39,40,41]. In addition, a sharp peak at 1362 cm^−1^, characteristic of the presence of NO_3_^−^ [42], was also identified.

Ultraviolet–visible (UV-Vis) spectral analysis revealed that, in comparison to H**L**, **[Ga(L)_2_]NO_3_** demonstrated distinct absorption bands at 420 nm (Figure 1b), which are characteristic of ligand-to-metal charge transfer (LMCT) transitions [41]. This spectral evidence confirms the successful formation of coordination bonds between the Ga(III) center and the organic ligand.

Energy dispersive X-ray spectroscopy (EDS) revealed an atomic ratio of Ga:S = 0.9:4.0 (equivalent to 1.0:4.4) for **[Ga(L)_2_]NO_3_** (Figure S4), consistent with the derived ratio of Ga:S ratio of 1.0:4.0 from the single-crystal data. X-ray photoelectron spectroscopy (XPS) of **[Ga(L)_2_]NO_3_** confirmed the presence of C, N, O, and Ga^3+^ (Figure S5). The Ga 3d XPS of **[Ga(L)_2_]NO_3_** showed two peaks at binding energies of 19.19 eV and 19.65 eV, which are assignable to the spin–orbit splitting of Ga 3d_5/2_ and Ga 3d_3/2_ of Ga^3+^ (Figure 1d) [43,44,45]. The N, N, S-conjugated tridentate moiety in the thiosemicarbazone ligand exhibited high electron cloud density and strong coordinating capability, potentially leading to a slight decrease in the binding energy of Ga 3d orbitals [43].

### 2.3. Fe^2+^ and Fe^3+^ Exchange with [Ga(L)_2_]NO_3_

**[Ga(L)_2_]NO_3_** smoothly underwent transmetalation with Fe^2+^ (using (NH_4_)_2_Fe(SO_4_)_2_·6H_2_O) and Fe^3+^ (using NH_4_Fe(SO_4_)_2_·12H_2_O) in DMF. For the transmetalation with Fe^2+^, the solution color gradually changed from light green to dark green (Figure S6), while for the corresponding reaction of Fe^3+^, the solution color gradually changed to yellowish-brown (Figure S7), presumably driven by the more stable complex formation. EDS of the exchanged samples indeed indicated that for both reactions, the Ga^3+^ was totally removed (Figure S8).

**Figure 2:**
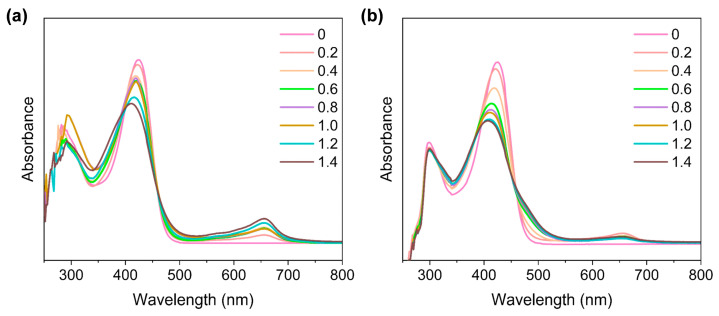
UV-Vis spectral changes observed during the titration of **[Ga(L)_2_]NO_3_** with (NH_4_)_2_Fe(SO_4_)_2_·6H_2_O (**a**) and with NH_4_Fe(SO_4_)_2_·12H_2_O (**b**). For each titration, a 1.0 equivalent solution of the Ga(III) complex in DMF was successively titrated with 0.2 equivalent aliquots of the respective iron salt.

The titration experiment for DMF-H_2_O indicated that for Fe^2+^ exchange, the LMCT band at 426 nm gradually shifted to 400 nm (Figure 2a), accompanied by the appearance of a new band at 644 nm, assignable as the lower energy charge transfer transition from the thiolate sulfur to Fe^2+^, which is unique to Fe(II)-TSC complexes [46]. It is interesting to note that, for the metathesis with Fe^3+^, the reaction initially generated Fe^2+^ species characterized by the generation of a low-energy peak at 657 nm (Figure 2b), presumably due to the reductive nature of the thiols [24,25]. Meanwhile, the LMCT band of **[Ga(L)_2_]NO_3_** gradually blue-shifted from 426 nm to 406 nm, accompanied by a shoulder peak at 484 nm, characteristic of Fe^3+^ bonding [47]. We further observed that the transmetalation reaction proceeded smoothly, as indicated by these titration curves and sample photographs recorded at 0.5 h, 6 h, and 72 h (Figures S6 and S7).

We further compared the exchange rate of **[Ga(L)_2_]NO_3_** with Fe^2+^/Fe^3+^. To achieve this, we reacted **[Ga(L)_2_]NO_3_** with an equivalent amount of Fe^2+^/Fe^3+^ at 60 °C in DMF/H_2_O, and monitored their absorbance changes at 654 nm over 24 h. As shown in Figure S9, both exchange reactions initially displayed first-order kinetics in the first three hours, with the exchange rate of Fe(III) being faster than that of Fe(II). Equilibrium was reached in around 7 h. Notably, the Fe(II) species formed were gradually oxidized, as seen in the slight decrease in the peak intensity at 24 h for both reactions.

In addition to Fe^2+^/Fe^3+^, **[Ga(L)_2_]NO_3_** was found to exhibit partial metal exchange with other ions as indicated by EDS, including Cu^2+^ (Cu:Ga = 1.0:0.6; exchange rate 63%; Figure S10a), Mn^2+^ (Mn:Ga = 0.1:0.5; exchange rate 17%; Figure S10b), Co^2+^ (Co:Ga = 0.7:0.1; exchange rate 88%; Figure S10c), Ni^2+^ (Ni:Ga = 1.0:0.5; exchange rate 67%; Figure S10d), and Zn^2+^ (Zn:Ga = 0.2:0.1; exchange rate 67%; Figure S10e). Nevertheless, given the inherently strong chelation characteristics of the H**L** ligand and the facile synthetic procedure, we suggest that these metal complexes can be most conveniently obtained by direct chelation.

### 2.4. Synthesis and Characterizations of [Ga(L)_2_]A Nanoparticles

Hyaluronic acid (HA) has been widely used for the delivery of drug molecules, exploiting the overexpression of the hyaluronan receptor CD-44 in numerous cancer cell lines [48,49,50]. Chemically, HA is rich in oxygen donors (carboxylate and hydroxyl) that can be useful for the delivery of metal-based medicines via binding to the metal ions. The sodium salt of HA, viz., NaA, in principle, can form stable nanoparticles with **[Ga(L)_2_]NO_3_** via the ejection of NaNO_3_. **[Ga(L)_2_]A** nanoparticles were prepared by stirring a DMSO solution of **[Ga(L)_2_]NO_3_** and an aqueous solution of NaA, followed by dialysis using a membrane with a molecular weight cutoff of 1000 Da. FT-IR spectroscopy showed that the peak at 1362 cm^−1^ (Figure S3), characteristic for NO_3_^−^, was absent in **[Ga(L)_2_]A**, indicating that the anion exchange process was complete [42].

**Figure 3:**
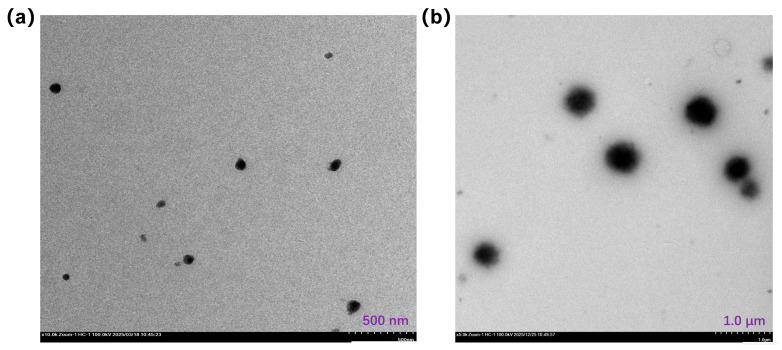
TEM images of **[Ga(L)_2_]A** (**a**) and particles formed from [Cu(NO_3_)(L)]_2_ + NaA (**b**).

Transmission electron microscopy (TEM) indicated that the particle sizes for **[Ga(L)_2_]A** were 82 ± 15 nm (Figure 3a), which is conducive to cellular uptake. The average hydrodynamic diameters of these particles, determined by dynamic light scattering (DLS), were measured to be 195.2 nm (PDI = 0.456; Figure S11). Such sizes measured by DLS are significantly larger than the corresponding TEM-derived values due to the hydration of the micelles [25,51]. The zeta potential for **[Ga(L)_2_]A** was −26.1 mV, which is significantly different from that of **[Ga(L)_2_]NO_3_** (3.09 mV) (Figure S12). Materials with neutral or positive zeta potentials generally exhibit enhanced cell binding affinity with cell membranes, although this may concurrently increase systemic toxicity [52]. Conversely, materials with negative zeta potentials, such as those described herein, are more likely to maintain prolonged plasma circulation, thereby optimizing enhanced cell permeability and retention (EPR) [53,54].

The advantage of ion pairs such as **[Ga(L)_2_]NO_3_** to the formation of hyaluronate-based particles is showcased by comparison of particle formation with our previously reported [Cu(NO_3_)(L)]_2_ featuring a coordinated NO_3_^−^ [22]. The reaction of [Cu(NO_3_)(L)]_2_ with NaA, following a similar protocol to that for **[Ga(L)_2_]A**, yielded much larger particles of around 440 nm (Figure 3b), and with partial replacement of NO_3_^−^ (Figure S13). This is probably due to the coordination of the carboxylate to the Cu(II) center to yield cross-linked structures with the dimeric [Cu(NO_3_)(L)]_2_.

We examined the stability of **[Ga(L)_2_]A** in serum-containing medium, i.e., 0.1 × (RPMI + 10% FBS + 1% P/S), as well as in deionized water, recorded over 0–72 h at room temperature by direct observation and UV-Vis spectroscopy to measure the absorbance intensity change at 600 nm (A600) (Figure S14) [55]. Aggregation of nanoparticles with a diameter of around 100 nm will cause a reduction in light transmission at λ = 600 nm due to increased light scattering by the colloids. A600-based turbidity monitoring is therefore a practical and commonly used measure to follow changes in nanoparticle dispersions over time. Within 72 h, the A600 of **[Ga(L)_2_]A** did not show a time-dependent increase in either serum-containing medium or in deionized water (Figure S14). For direct observation, we intentionally used a more concentrated dispersion to make any potential agglomeration easier to observe. Again, there was no obvious agglomeration for these samples in the 0.1 × (RPMI + 10% FBS + 1% P/S) solution and deionized water. We therefore conclude that these particles are stable in serum-containing medium or deionized water and do not aggregate over time.

### 2.5. Cell Cytotoxicity Assay

**Figure 4:**
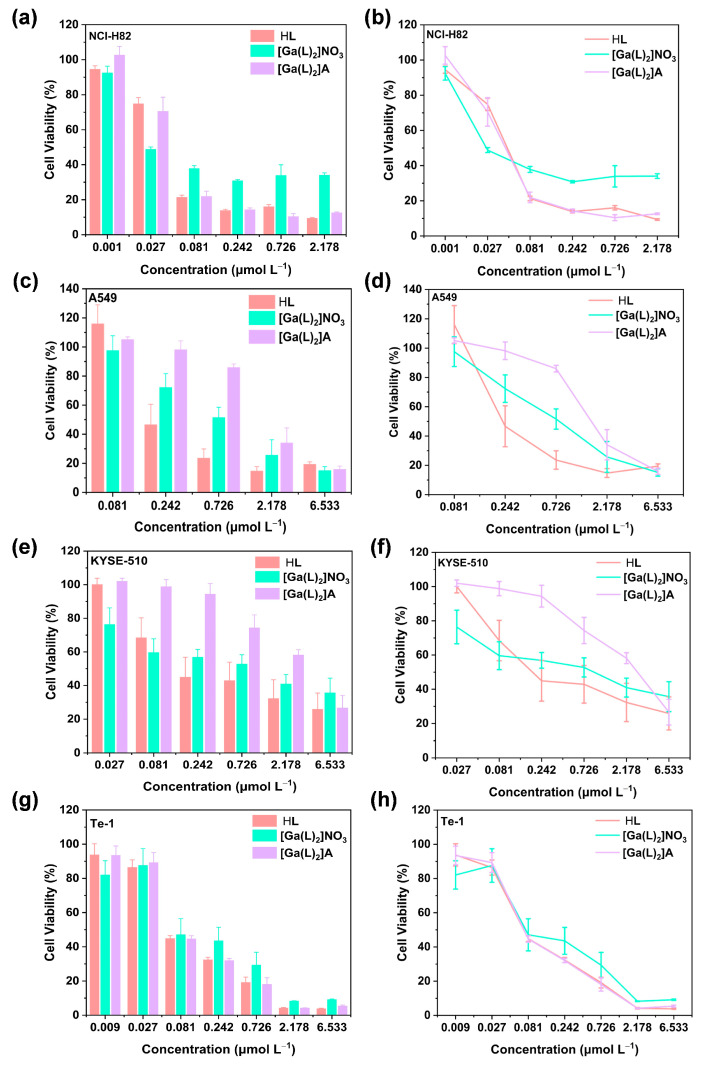
A comparison of cell viability of NCI-H82 (**a**,**b**), A549 (**c**,**d**), KYSE-510 (**e**,**f**), and Te-1 (**g**,**h**) upon incubation with gradient concentrations H**L**, **[Ga(L)_2_]NO_3_**, and **[Ga(L)_2_]A**.

To evaluate the anticancer potency of **[Ga(L)_2_]A**, we assessed their inhibitory rates against four cancer cell lines: NCI-H82 (lung cancer), A549 (lung cancer), KYSE-510 (esophageal cancer), and Te-1 (esophageal cancer), and compared them with those for H**L** and **[Ga(L)_2_]NO_3_**. As shown in Figure 4a–h, both **[Ga(L)_2_]NO_3_**, and **[Ga(L)_2_]A** exhibited superior in vitro cytotoxicity against all these cell lines, featuring half maximal inhibitory concentration (IC_50_) values in the range of 0.102–2.616 μmol L^−1^ for **[Ga(L)_2_]A** (Table 3). These values are comparable to other Ga^3+^- and Fe^3+^-based TSC complexes reported in the literature, as well as the benchmarking drugs used clinically, such as DOX and cisplatin [56,57], indicating the potency of **[Ga(L)_2_]A** in killing diverse cancer cells.

It is also notable that comparing H**L**, **[Ga(L)_2_]NO_3_**, and **[Ga(L)_2_]A**, the free ligand H**L** exhibited the highest toxicity, followed by **[Ga(L)_2_]NO_3_** and **[Ga(L)_2_]A**. This is presumably due to their different working mechanisms (i.e., different cellular uptake processes between small molecular drugs and nanoparticles, direct metal sequestration of the free ligand H**L**, and the necessity of metathesis for metal-based drugs).

### 2.6. Cellular Uptake of [Ga(L)_2_]A

To evaluate the contribution of hyaluronate in the cellular uptake process, we pretreated KYSE-510 cells with NaA followed by a dosage of **[Ga(L)_2_]A** (NaA-pretreated cells are denoted as **[Ga(L)_2_]A′**), and compared the results with those of directly administered **[Ga(L)_2_]A**. As shown in Table 4, the inductively coupled plasma mass spectrometric data (ICP-MS) showed that drug uptake by the cells increased with incubation time. However, the uptake of **[Ga(L)_2_]A** by KYSE-510 cells pretreated with NaA was lower than that of cells directly treated with **[Ga(L)_2_]A**. These results indicate that the drug **[Ga(L)_2_]A** modified by hyaluronic acid has a CD-44 targeting effect.

## 3. Materials and Methods

### 3.1. General

Ligand H**L** and [Cu(NO_3_)(L)]_2_ were synthesized as described in our previous report [22]. Ga(NO_3_)_3_·9H_2_O (99.99%, Xiya Chemical Technology Co., Ltd., Shandong, China), (NH_4_)_2_Fe(SO_4_)_2_·6H_2_O (99.5%, Shanghai Lingfeng Chemical Reagent Co., Ltd., Shanghai, China), NH_4_Fe(SO_4_)_2_·12H_2_O (99%, Macklin Biochemical Co., Ltd., Shanghai, China), Co(CH_3_COO)_2_ (98%, Shanghai Acmec Biochemical Co., Ltd., Shanghai, China), ZnCl_2_ (99.95%, Shanghai Aladdin Biochemical Technology Co., Ltd., Shanghai, China), NiBr_2_·*x*H_2_O (98%, Adamas Reagent Co., Ltd., Shanghai, China), Mn(CH_3_COO)_2_·4H_2_O (98%, Shanghai Titan Scientific Co., Ltd., Shanghai, China) and sodium hyaluronate (97%, Energy Chemicals, Shanghai, China) were available from the corresponding suppliers without further purification. CH_2_Cl_2_, CH_3_OH, N, N-dimethylformamide (DMF), dimethyl sulfoxide (DMSO), and diethyl ether (Et_2_O), all of analytical grade, were procured from Chinasun Specialty Products Co., Ltd. (Jiangsu, China).

Cell lines NCI-H82, A549, KYSE-510, and Te-1 were purchased from the Shanghai Institute of Cell Biology, Chinese Academy of Sciences (Shanghai, China). 0.25% Trypsin solution (containing EDTA, dissolved in PBS) was purchased from Procell Life Science & Technology Co., Ltd. (Hubei, China) Cell culturing medium RPMI 1640 (10% FBS + 1% P/S), RPMI 1640 (1% P/S), Ham’s F-12 (10% FBS + 1% P/S), Ham’s F-12 (1% P/S), and Phosphate-buffered solution (PBS) were purchased from Shanghai Basal Media Technologies Co., Ltd. (Shanghai, China) The cell counting kit-8 (CCK-8) was purchased from APExBIO Technology LLC (Shanghai, China).

^1^H nuclear magnetic resonance (NMR) spectra were recorded on a BRUKER AVANCE III HD 400 MHz superconducting NMR spectrometer (Bruker AXS GmbH, Karlsruhe, Germany). Fourier-transform infrared (FT-IR) spectra were measured using a Bruker VERTEX 70 + HYPERION 2000 FT-IR spectrometer (Bruker AXS GmbH, Karlsruhe, Germany), employing the attenuated total reflection (ATR) technique. Ultraviolet–visible (UV-Vis) spectra were acquired using a Varian Cary-50 UV-Vis spectrophotometer (Varian, Inc., Palo Alto, CA, USA). X-ray photoelectron spectroscopy (XPS) was performed on an EXCALAB 250 XI X-ray photoelectron spectrometer (Thermo Scientific, Waltham, MA, USA). Energy-dispersive X-ray spectroscopy (EDS) was conducted using a ZEISS EVO 18 scanning electron microscope (ZEISS Group, Oberkochen, Germany). Transmission electron microscopy (TEM) images were obtained using a HITACHI HT7700 transmission electron microscope (Hitachi, Tokyo, Japan), and samples were prepared by dropping aqueous solutions onto copper grids. Dynamic light scattering (DLS) and zeta potential measurements were performed using an LA-950S2 laser particle size analyzer (Horiba, Kyoto, Japan). The high-performance liquid chromatography–mass spectrometry (HPLC-MS) was carried out on an Agilent 1260 Infinity II Bio-SEC system (Agilent Technologies, Inc., Santa Clara, CA, USA). Inductively coupled plasma-mass spectrometry (ICP-MS) was performed with an iCAP PRO instrument (Thermo Scientific, Waltham, MA, USA). Cytotoxicity assays were performed on a TECAN M1000 PRO microplate reader (Tecan, Zürich, Switzerland) by measuring absorbance at 450 nm.

### 3.2. Synthesis of [Ga(L)_2_]NO_3_

Ga(NO_3_)_3_·9H_2_O (2.5 mg, 6.0 μmol) and H**L** (3.0 mg, 7.3 μmol) were added to a 10 mL centrifuge tube. CH_3_OH (3 mL) was then added dropwise under ambient conditions to achieve complete dissolution. The reaction mixture was stirred at room temperature for 24 h, followed by centrifugation to remove the precipitate. The clear yellow solution was diffused with anhydrous ether to give yellow block crystals of **[Ga(L)_2_]NO_3_**·as the CH_3_OH solvate after 7 days. The crystals were filtered, washed with anhydrous ether, and dried. Yield: 0.8 mg (23.3% based on H**L**). IR (ATR, cm^−1^): 3360(m), 2920(s), 2850(m), 1633(w), 1598(m), 1513(s), 1488(s), 1463(s), 1425(vs), 1362(vs), 1336(s), 1299(vs), 1276(vs), 1237(vs), 1224(vs), 1202(vs), 1173(s), 1133(vs), 1097(s), 1057(s), 1009(vs), 980(s), 956(m), 912(s), 870(w), 854(w), 823(s), 792(s), 746(s), 725(m), 696(w), 661(m), 646(m), 613(m). ^1^H NMR (400 MHz, DMSO-*d*_6_) δ 8.89 (d, *J* = 3.8 Hz, 2H), 8.32 (d, *J* = 7.7 Hz, 2H), 8.26–8.11 (m, 6H), 7.84 (d, *J* = 8.0 Hz, 2H), 7.76–7.66 (m, 4H), 7.18 (d, *J* = 3.2 Hz, 2H), 6.89 (d, *J* = 3.2 Hz, 2H), 3.94 (s, 8H), 3.49 (s, 8H).

### 3.3. Single-Crystal X-Ray Crystallography

Diffraction data were acquired on a Bruker APEX II CCD X-ray diffractometer (Bruker AXS GmbH, Karlsruhe, Germany) using Mo-Kα (λ = 0.71073 Å) irradiation for **[Ga(L)_2_]NO_3_**. Refinement of and reduction in the collected data were achieved using the program SAINT, and absorption corrections were performed using a multi-scan method [64]. The crystal structures of **[Ga(L)_2_]NO_3_** were solved by direct methods and refined on *F*^2^ by full-matrix least-squares techniques with SHELXTL-2016 [65].

Crystallographic data for **[Ga(L)_2_]NO_3_** have been deposited in the Cambridge Crystallographic Data Center (CCDC) as supplementary publication number 2482780. These data can be obtained free of charge either from the CCDC via www.ccdc.cam.ac.uk/data_request/cif (accessed on 10 October 2025), or from the Supplementary Information. A summary of the key crystallographic data for **[Ga(L)_2_]NO_3_** is listed in Table 1.

### 3.4. Fe^2+^ Exchange with [Ga(L)_2_]NO_3_

**[Ga(L)_2_]NO_3_** (3.0 mg, 3.2 μmol) and (NH_4_)_2_Fe(SO_4_)_2_·6H_2_O (1.2 mg, 3.2 μmol) were dissolved in 5 mL of DMF, and the mixture was stirred at room temperature for 72 h. The solution color gradually changed from orange-yellow to dark green. A portion of 50 mL H_2_O was then introduced, and the solution was extracted three times with 80 mL CH_2_Cl_2_. The CH_2_Cl_2_ layers were then combined, and the solvent was removed by rotary evaporation to give the yellowish-brown powder.

### 3.5. Fe^3+^ Exchange with [Ga(L)_2_]NO_3_

**[Ga(L)_2_]NO_3_** (3.0 mg, 3.2 μmol) and NH_4_Fe(SO_4_)_2_·12H_2_O (1.5 mg, 3.2 μmol) were dissolved in 5 mL of DMF, and the mixture was stirred at room temperature for 72 h. The solution changed from orange-yellow to yellowish-brown. A portion of 50 mL H_2_O was then introduced, and the solution was extracted three times with 80 mL CH_2_Cl_2_. The CH_2_Cl_2_ layers were then combined, and the solvent was removed by rotary evaporation to give the yellowish-brown powder.

### 3.6. Ion Exchange of [Ga(L)_2_]NO_3_ with Different Transition Metal Ions

**[Ga(L)_2_]NO_3_** (10.0 mg, 10.56 μmol) and an equivalent of the respective metal salt Cu(NO_3_)_2_·*x*H_2_O, Mn(CH_3_COO)_2_·4H_2_O, Co(CH_3_COO)_2_, NiBr_2_·*x*H_2_O, or ZnCl_2_ were dissolved in 10 mL of CH_3_OH, and the mixture was stirred at room temperature for 72 h. The CH_2_Cl_2_ phase was extracted with water three times, and the solvent was then removed by rotary evaporation to afford the respective ion exchange products subjected to EDS analysis.

### 3.7. Exchange Rate Comparison Between [Ga(L)_2_]NO_3_ and Fe^2+^/Fe^3+^

A DMF solution of **[Ga(L)_2_]NO_3_** with a concentration of 3.17 × 10^−4^ mol·L^−1^ (10.0 mL) was prepared. An aqueous solution of (NH_4_)_2_Fe(SO_4_)_2_·6H_2_O (1.26 × 10^−2^ mol·L^−1^) was then added to achieve an equimolar ratio of Fe(II) to **[Ga(L)_2_]NO_3_**. The reaction mixture was maintained at 60 °C, and aliquots were withdrawn at 0, 2, 10, 20, 40, 60, 120, 240, 360, 480, 600, and 1440 min. Each aliquot was diluted 10-fold with DMF before measurement, and the time-dependent spectral changes at 654 nm were monitored by UV-Vis absorption spectroscopy. Titration experiments with Fe^3+^ were carried out in the same manner using NH_4_Fe(SO_4_)_2_·12H_2_O.

### 3.8. Stability Analysis of [Ga(L)_2_]A

**[Ga(L)_2_]A** was respectively dispersed in 0.1 × (RPMI + 10% FBS + 1% P/S) solution and in deionized water. Each solution was gently shaken and then stored at room temperature under static conditions. Aliquots were collected at different time intervals (0, 24, 48, and 72 h) to record the absorbance at λ = 600 nm (A600), using the corresponding 0.1 × (RPMI + 10% FBS + 1% P/S) solution or deionized water as the blank.

### 3.9. Titration Experiment

For the titration with Fe^2+^, a DMF solution of **[Ga(L)_2_]NO_3_** with a concentration of 1.06 × 10^−3^ mol·L^−1^ and an aqueous solution of (NH_4_)_2_Fe(SO_4_)_2_·6H_2_O with a concentration of 1.28 × 10^−2^ mol·L^−1^ were prepared as the titrant. Eight aliquots (3 mL each) of the complex solution were treated with different volumes of the Fe^2+^ titrant to achieve Fe^2+^ equivalent ratios of 0, 0.2, 0.4, 0.6, 0.8, 1.0, 1.2, and 1.4 eq relative to the complex. After reacting at room temperature for 0.5 h, 6 h, and 72 h, respectively, each reaction mixture was diluted 40 times with DMF, and its spectral changes were monitored by UV-Vis spectroscopy. The titration experiments with Fe^3+^ were carried out in the same manner using NH_4_Fe(SO_4_)_2_·12H_2_O.

### 3.10. Nanoparticle Formations of [Ga(L)_2_]A

**[Ga(L)_2_]NO_3_** (10.0 mg, 10.6 μmol) was dissolved in DMSO (1 mL) and then added dropwise to an aqueous solution of sodium hyaluronate (68.8 mg in 11 mL of water). The mixture was stirred at r.t. and protected from light for 72 h to obtain the resulting nanoparticles, which were dialyzed for 24 h (molecular weight cut-off of 1000 Da) to obtain **[Ga(L)_2_]A**. The preparation process for Cu-based particles was similar except that [Cu(NO_3_)(L)]_2_ [22] was used instead of **[Ga(L)_2_]NO_3_**.

### 3.11. Determination of the Concentration of [Ga(L)_2_]A

The concentration of **[Ga(L)_2_]A** was determined by UV-Vis spectroscopy. A calibration curve was established using **[Ga(L)_2_]NO_3_** as the reference. A series of standard aqueous solutions with different concentrations was prepared, and their absorbance values at 420 nm (LMCT band) were recorded. A linear relationship between the absorbance and concentration was observed within the tested concentration range (Figure S15). The aqueous dispersion of **[Ga(L)_2_]A** was measured under identical conditions, and its absorbance at 420 nm was converted to concentration using the calibration curve. As each **[Ga(L)_2_]^+^** unit contains a pair of ligands, the calculated **[Ga(L)_2_]^+^** concentration was multiplied by two to obtain the ligand-equivalent concentration, which was used consistently for dose definition in all biological experiments.

### 3.12. In Vitro Cytotoxicity Evaluation by CCK-8 Assay

The NCI-H82 cell line (suspension-grown) was cultured in RPMI 1640 medium supplemented with 10% FBS, 1% P/S, or RPMI 1640 medium containing 1% P/S. Specifically, the suspension-grown cells were centrifuged, and the supernatant was discarded. The cells were resuspended in serum-supplemented RPMI 1640 medium at a concentration of 1 × 10^5^ cells mL^−1^. These cells were cultured at 37 °C under a 5% CO_2_ atmosphere for the CCK-8 assays.

NCI-H82 cells were seeded at a density of 2 × 10^4^ cells per well in 100 µL of serum-free medium (RPMI 1640 + 1% P/S) containing various concentrations of H**L**, **[Ga(L)_2_]NO_3_**, **[Ga(L)_2_]A**. All experiments were conducted with five replicates (*n* = 5), using untreated cells as the 100% cell viability control and cell-free medium (RPMI 1640 + 10 µL FBS + 1% P/S + CCK-8) as the blank.

The NCI-H82 cells were incubated continuously for 72 h. After the incubation period, 10 µL FBS and 10 µL CCK-8 were added to the wells, and the plates were further incubated for 3.5 h before analysis at 450 nm using a microplate reader. The relative cell viability (%) was calculated using Equation (1) as described below:

(1) $$V\%=\frac{{\left[A\right]}_{experimental}-{\left[A\right]}_{blank}}{{\left[A\right]}_{control}-{\left[A\right]}_{blank}}\times 100\%$$

in which V% is the percentage of cell viability, [*A*]*_experimental_* is the absorbance of the wells culturing the treated cells, [*A*]*_blank_* is the absorbance of the blank, and [*A*]*_control_* is the absorbance of the wells culturing untreated cells.

The A549 cell line (adherent cells) was cultured in Ham’s F-12 medium supplemented with 10% FBS, 1% P/S, or Ham’s F-12 medium containing 1% P/S. Specifically, the adherent cells grew as a monolayer and were detached at confluence using trypsin (0.5% *w*/*v* in PBS). After trypsinization, the cells were incubated for 3 min, centrifuged, and the supernatant was discarded. A 3 mL portion of serum-supplemented culture medium was added to neutralize any residual trypsin. The cells were re-suspended in serum-supplemented Ham’s F-12 medium at a concentration of 1 × 10^5^ cells mL^−1^ and cultured under standard conditions (37 °C, 5% CO_2_) for the CCK-8 studies.

A549 cells were seeded at a density of 1 × 10^4^ cells per well in 90 µL of culture medium (Ham’s F-12 + 10% FBS + 1% P/S) and cultured for 24 h at 37 °C and 5% CO_2_ to allow cell attachment. The culture medium was then replaced with serum-free medium (Ham’s F-12 + 1% P/S) containing various concentrations of the H**L**, **[Ga(L)_2_]NO_3_**, **[Ga(L)_2_]A**. All experiments were performed with five replicates (*n* = 5), using untreated cells as the 100% cell viability control and cell-free medium (Ham’s F-12 + 10% FBS + 1% P/S + CCK-8) as the blank.

The A549 cells were incubated continuously for 72 h. After the incubation period, 10 µL of FBS and 10 µL of CCK-8 were added to each well, and the plates were incubated for an additional 2.5 h before being analyzed at 450 nm using a microplate reader. The relative cell viability (%) was calculated using Equation (1) above.

For the KYSE-510 (adherent cells) and Te-1 (adherent cells) cell lines, the cytotoxicity assessment followed a similar protocol to that of the A549 cells, with the exception of the culture media. KYSE-510 cells were cultured in a 1:1 mixture of RPMI 1640 and Ham’s F-12 media, while Te-1 cells were cultured in RPMI 1640 medium. The exact compositions were: RPMI 1640:Ham’s F-12 = 1:1 + 10% FBS + 1% P/S and RPMI 1640:Ham’s F-12 = 1:1 + 1% P/S for KYSE-510; RPMI 1640 + 10% FBS + 1% P/S and RPMI 1640 + 1% P/S for Te-1.

### 3.13. Cellular Uptake

KYSE-510 cells were seeded into six 10 cm cell culture dishes at a density of 2 × 10^5^ cells per dish. Cells were cultured in serum-supplemented medium (RPMI 1640:Ham’s F-12 = 1:1 + 10% FBS + 1% P/S) until reaching approximately 95% confluence. The medium was then replaced with serum-free medium. To distinguish the group pretreated with sodium hyaluronate from the direct treatment group, designated **[Ga(L)_2_]A**, the pretreated group was labeled **[Ga(L)_2_]A′**. For the pretreated group, dishes were pretreated with 30 mg of sodium hyaluronate for 24 h prior to the addition of **[Ga(L)_2_]A**, while the **[Ga(L)_2_]A** group was administered the drug directly. Both groups were exposed to **[Ga(L)_2_]A** at a final H**L** concentration of 50 μM. Cells were incubated with the compound for 2 h, 4 h, or 6 h. Following incubation, cells were washed three times with PBS buffer and harvested via trypsin digestion. The harvested cells were collected into 15-mL centrifuge tubes and centrifuged at 900 rpm for 3 min. The resulting pellet was washed twice with PBS and centrifuged again. For metal content analysis, the cell pellet was digested with 0.4 mL concentrated nitric acid. The digest was diluted to 3 mL with deionized water, filtered through a 0.45 μm membrane, and subjected to ICP-MS analysis to quantify the Ga^3+^ concentration.

## 4. Conclusions

Intrigued by the biocompatibility of Ga^3+^ and the similarities between Ga^3+^ and Fe^3+^, we have demonstrated that the thiosemicarbazone ligand H**L** can form a biologically active, cationic octahedral complex **[Ga(L)_2_]NO_3_**, and that nitrate-for-hyaluronate surrogation further yielded **[Ga(L)_2_]A** as a nanomedicine that endowed the material with aqueous solubility and cell selectivity. The facile yet gradual metathesis of **[Ga(L)_2_]NO_3_** with both Fe^2+^ and Fe^3+^ suggests that **[Ga(L)_2_]NO_3_** could be a promising anticancer drug with ‘stealth’ properties during drug delivery. Further investigation of the mechanisms of action, oral bioavailability, and in vivo performance is needed in pursuit of these goals.

## Figures and Tables

**Table 1:** Crystal data and structure refinement parameters for **[Ga(L)_2_]NO_3_**.

	[Ga(L)_2_]NO_3_
Formula	C_39_H_40_GaN_15_O_4_S_4_
Formula Weight	980.82
Crystal System	Monoclinic
Space Group	*P*2_1_*/n*
*a*/Å	13.7231(4)
*b*/Å	16.5511(5)
*c*/Å	19.3202(7)
*β*/°	93.1060(10)
*V*/Å^3^	4381.8(2)
*Z*	4
*ρ*_calc_/(g cm^−3^)	1.487
*F*(000)	2024
*μ* (Mo–Kα)/mm^−1^	0.879
Total Reflections	102,760
Unique Reflections	10,031
No. Observations	8243
*R_int_*	0.0528
No. Parameters	570
*R ^a^*	0.0374
*wR ^b^*	0.0912
*GOF ^c^*	1.111

*^a^* *R*_1_ = Σ||*F*_o_| − |*F*_c_||/Σ|*F*_o_|, *^b^ wR*_2_ = {Σ[*w*(*F*_o_^2^ − *F*_c_^2^)^2^]/Σ[*w*(*F*_o_^2^)^2^]}^1/2^, *^c^ GOF* = {Σ[*w*(*F*_o_^2^ − *F*_c_^2^)^2^]/(*n* − *p*)}^1/2^, where *n* is the number of reflections and *p* is total number of parameters refined.

**Table 2:** Selected bond distances (Å) and angles (°) of **[Ga(L)_2_]NO_3_** involving the Ga(III) center.

Ga1–S1	2.3600(6)	Ga1–S3	2.3715(6)
Ga1–N12	2.0493(17)	Ga1–N5	2.0529(16)
Ga1–N7	2.0938(18)	Ga1–N13	2.1249(18)
N12–Ga1–N5	175.06(7)	N12–Ga1–N7	97.70(7)
N5–Ga1–N7	78.03(7)	N12–Ga1–N13	77.30(7)
N5–Ga1–N13	99.88(7)	N7–Ga1–N13	87.69(7)
N12–Ga1–S1	101.96(5)	N5–Ga1–S1	82.07(5)
N7–Ga1–S1	159.62(5)	N13–Ga1–S1	91.44(5)
N12–Ga1–S3	82.12(5)	N5–Ga1–S3	100.30(5)
N7–Ga1–S3	90.52(5)	N13–Ga1–S3	158.91(5)
S1–Ga1–S3	97.36(2)		

**Table 3:** A comparison of the IC_50_ values of H**L**, **[Ga(L)_2_]NO_3_**, and **[Ga(L)_2_]A** with literature examples for different cell lines.

Entry	Compound	Cell Line	IC_50_ (µM)	Reference
1	DOX	A549	0.058	[56]
2	Cisplatin	A549	36.39	[57]
3	[Ga(L_a_)_2_]NO_3_	RT2	810	[58]
4	[Ga(L_b_)_2_(NO_3_)]·*x*H_2_O	HCT-116	0.55	[59]
5	[Ga(L_c_)_2_]PF_6_	SK-BR-3	1.7 × 10^−4^	[6]
6	Ga(L_d_)Cl_2_	MCF-7	1.05	[60]
7	[Fe(L_e_)_2_](ClO_4_)	SK-N-MC	0.19	[61]
8	Fe(L_f_)_2_(NO_3_)(H_2_O)_3_	SW-480	19.11	[47]
9	[Fe(L_g_)_2_](ClO_4_)	HL60	0.4	[62]
10	[Fe(L_h_)_2_]Cl	MDA-MB-231	12.38	[63]
11	[Cu(NO_3_)(L)]_2_	Hep-G2	16.86	[22]
12	H**L**	NCI-H82	0.041	This work
13	H**L**	A549	0.107	This work
14	H**L**	KYSE-510	0.095	This work
15	H**L**	Te-1	0.108	This work
16	**[Ga(L)_2_]NO_3_**	NCI-H82	0.029	This work
17	**[Ga(L)_2_]NO_3_**	A549	0.787	This work
18	**[Ga(L)_2_]NO_3_**	KYSE-510	0.463	This work
19	**[Ga(L)_2_]NO_3_**	Te-1	0.176	This work
20	**[Ga(L)_2_]A**	NCI-H82	0.102	This work
21	**[Ga(L)_2_]A**	A549	1.342	This work
22	**[Ga(L)_2_]A**	KYSE-510	2.616	This work
23	**[Ga(L)_2_]A**	Te-1	0.267	This work

DOX = doxorubicin; L_a_ = N(4)-methyl-2-pyridineformamide thiosemicarbazone; L_b_ = N^1^-(7-chloro-quinolin-4-yl)-ethylamino-2-acetylpyridine-thiosemicarbazone; L_c_ = acetylpyrazine N, N-dimethylthiosemicarbazone; L_d_ = isopropyl-2-pyridyl-ketone-4, 4-dimethylthiosemicarbazone; L_e_ = (Z)-3-phenyl-1-(2-pyridinyl)-2-propen-1-one-4, 4-dimethyl-3-thiosemicarbazone; L_f_ = pyridine-2-carbaldehyde 4-N-methylthiosemicarbazone; L_g_ = dipyridylketone 4-allylthiosemicarbazone; L_h_ = 2-benzoylpyridine 4-methyl-3-thiosemicarbazone.

**Table 4:** Cellular uptake of **[Ga(L)_2_]A** by KYSE-510 (1 × 10^7^ cells) with and without NaA pre-treatment, as determined by Ga^3+^ concentration (ppb) using ICP-MS. The NaA pre-treated sample is denoted as **[Ga(L)_2_]A′**.

	[Ga(L)_2_]A	[Ga(L)_2_]A′
2 h	310.325	224.521
4 h	503.897	309.679
6 h	554.22	323.187

## Data Availability

Data are contained within the article and Supplementary Materials.

## Supplementary Materials

The following supporting information can be downloaded at: https://www.mdpi.com/article/10.3390/molecules31030577/s1, Additional structure diagrams, spectroscopies, and a hydrogen-bonding table.
