# Supplementary material for: The Synthesis, Metal Exchange, and Hyaluronate Functionalization of a Cationic Gallium-Based Thiosemicarbazone Anticancer Drug
Source: Molecules. 2026 Feb 6;31(3):577. doi: 10.3390/molecules31030577 (PMC12899563; doi:10.3390/molecules31030577)
Supplement: Supplementary file 1 [file molecules-31-00577-s001.zip › molecules-4057175-supplementary.pdf]

Supplementary Materials

Content

Figure S1 Crystal packing diagram of **$[Ga(L)_2]NO_3$** looking along the crystallographic *a* axis. Hydrogen-bonding interactions between the MeOH solvate and NO_3^- are shown as dashed lines. Except for that of MeOH, all the hydrogen atoms are omitted for clarity. Color legend: Ga (dark green), S (yellow), O (red), N (blue), C (black), and H (light pink). 3
Figure S2 The ^1H NMR spectra of H**L** (a) and **$[Ga(L)_2]NO_3$** in DMSO-d_6 (b). 4
Figure S3 FT-IR spectra of NaA, H**L**, **$[Ga(L)_2]NO_3$**, and **$[Ga(L)_2]A$** in the 4000–600 cm^{-1} region (a), and the enlarged view of (a) in the 1800–1200 cm^{-1} region (b). 5
Figure S4 The EDS spectra of **$[Ga(L)_2]NO_3$**. 6
Figure S5 The full XPS spectra of **$[Ga(L)_2]NO_3$**. 7
Figure S6 UV-Vis spectral and color (insets) changes of **$[Ga(L)_2]NO_3$** upon titration with Fe^{2+}, monitored at 0.5 h (a), 6 h (b), and 72 h (c). 8
Figure S7 UV-Vis spectral and color (insets) changes of **$[Ga(L)_2]NO_3$** upon titration with Fe^{3+}, monitored at 0.5 h (a), 6 h (b), and 72 h (c). 9
Figure S8 The EDS spectra of the solid products obtained from the reaction of **$[Ga(L)_2]NO_3$** with Fe^{2+} (a) and Fe^{3+} (b). 10
Figure S9 Time-resolved UV–Vis spectra of **$[Ga(L)_2]NO_3$** reacting with $(NH_4)_2Fe(SO_4)_2·6H_2O$ (a) and $NH_4Fe(SO_4)_2·12H_2O$ (b) at an molar ratio of 1 : 1 in DMF/H_2O at 60 °C (0–24 h). Theoretical fitting curves of the absorbance at 654 nm for (a) and (b) at different time intervals (c). 11
Figure S10 The EDS spectra of the solid products obtained from the reaction of **$[Ga(L)_2]NO_3$** with $Cu(NO_3)_2·xH_2O$ (a), $Mn(CH_3COO)_2·4H_2O$ (b), $Co(CH_3COO)_2$ (c), $NiBr_2·xH_2O$ (d), and $ZnCl_2$ (e), showing the incorporation of different transition metal ions. 14
Figure S11 The DLS spectra of **$[Ga(L)_2]A$**. 15
Figure S12 The zeta potential diagrams of **$[Ga(L)_2]NO_3$** (a) and **$[Ga(L)_2]A$** (b). 16
Figure S13. A comparison of the FT-IR spectra of H**L**, **$[Cu(NO_3)(L)]_2$**, **$[Cu(NO_3)(L)]_2$** + NaA, and NaA. 17
Figure S14 Turbidity-based stability assessment of **$[Ga(L)_2]A$** dispersions at the absorbance of 600 nm in 0.1 × (RPMI + 10% FBS + 1% P/S) and deionized water recorded at 0, 24, 48, and 72 h at room temperature (a). Photographs of **$[Ga(L)_2]A$** dispersions stored in 0.1 × (RPMI + 10% FBS + 1% P/S) and deionized water for 0, 24, 48, and 72 h at room temperature (b). . 18
Figure S15 Standard curve of **$[Ga(L)_2]NO_3$** in deionized water. The plot depicts the absorbance at 420 nm as a function of concentration, showing a linear relationship in accordance with the Beer-Lambert law. 19
Table S1. Hydrogen bonding interactions for **$[Ga(L)_2]NO_3$** [Å and °]. 20

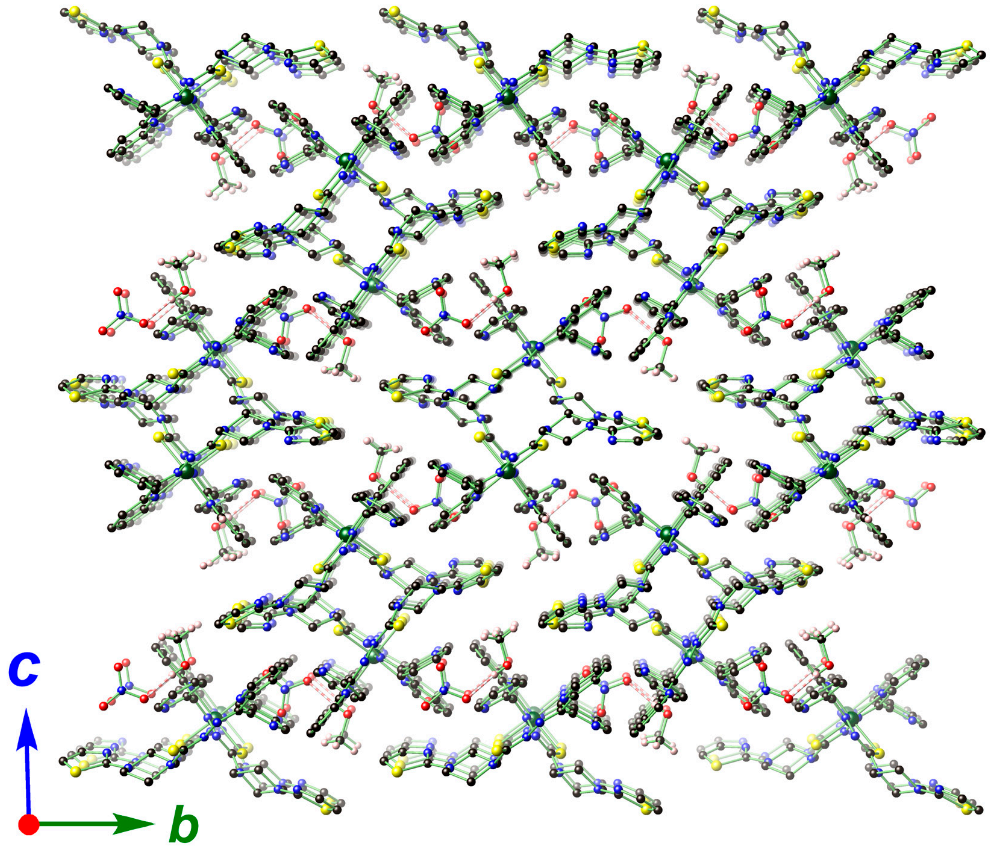

Figure S1 Crystal packing diagram of **[Ga(L)$_2$]NO$_3$** looking along the crystallographic *a* axis. Hydrogen-bonding interactions between the MeOH solvate and NO_3^- are shown as dashed lines. Except for that of MeOH, all the hydrogen atoms are omitted for clarity. Color legend: Ga (dark green), S (yellow), O (red), N (blue), C (black), and H (light pink).

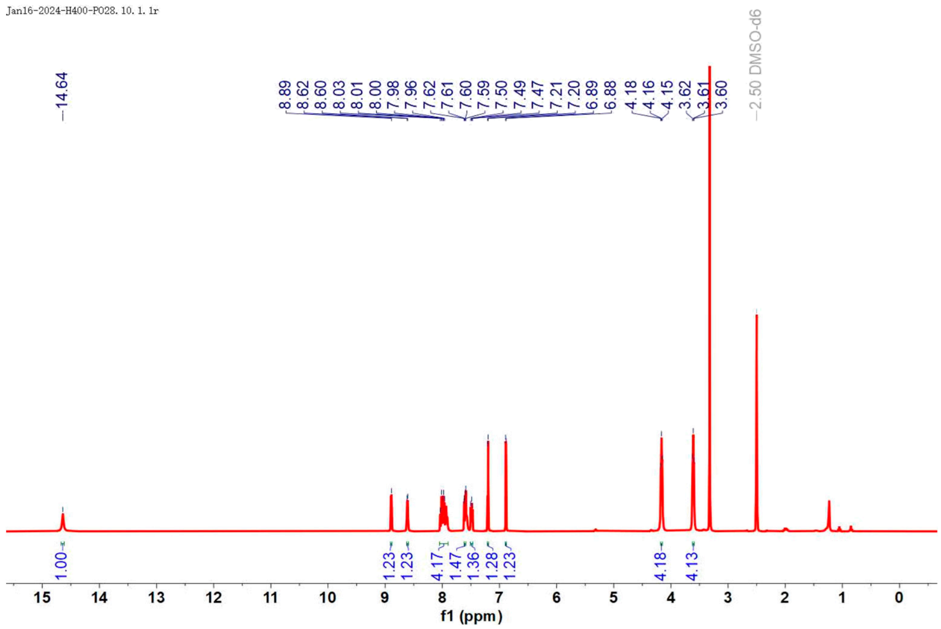

(a)

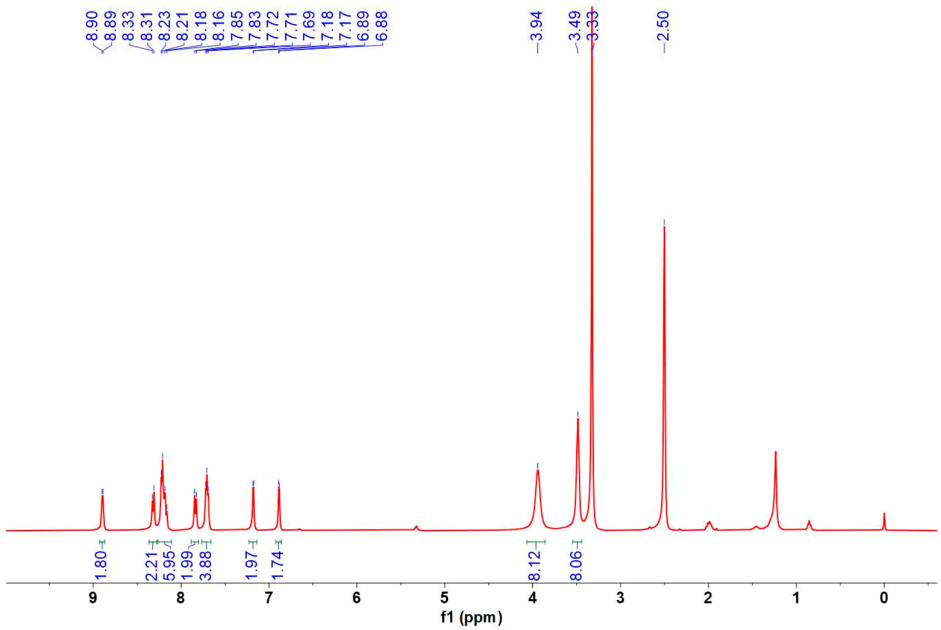

(b)

Figure S2 The ^{1}H NMR spectra of H**L** (a) and **[Ga(L)$_2$]NO$_3$** in DMSO-d_6 (b).

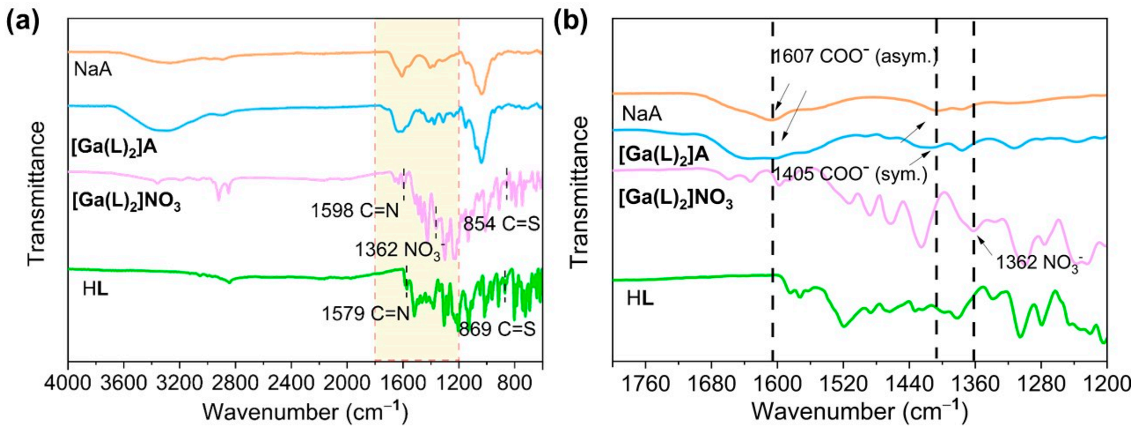

Figure S3 FT-IR spectra of NaA, H**L**, **[Ga(L)$_2$]NO$_3$**, and **[Ga(L)$_2$]A** in the 4000–600 cm^{-1} region (a), and the enlarged view of (a) in the 1800–1200 cm^{-1} region (b).

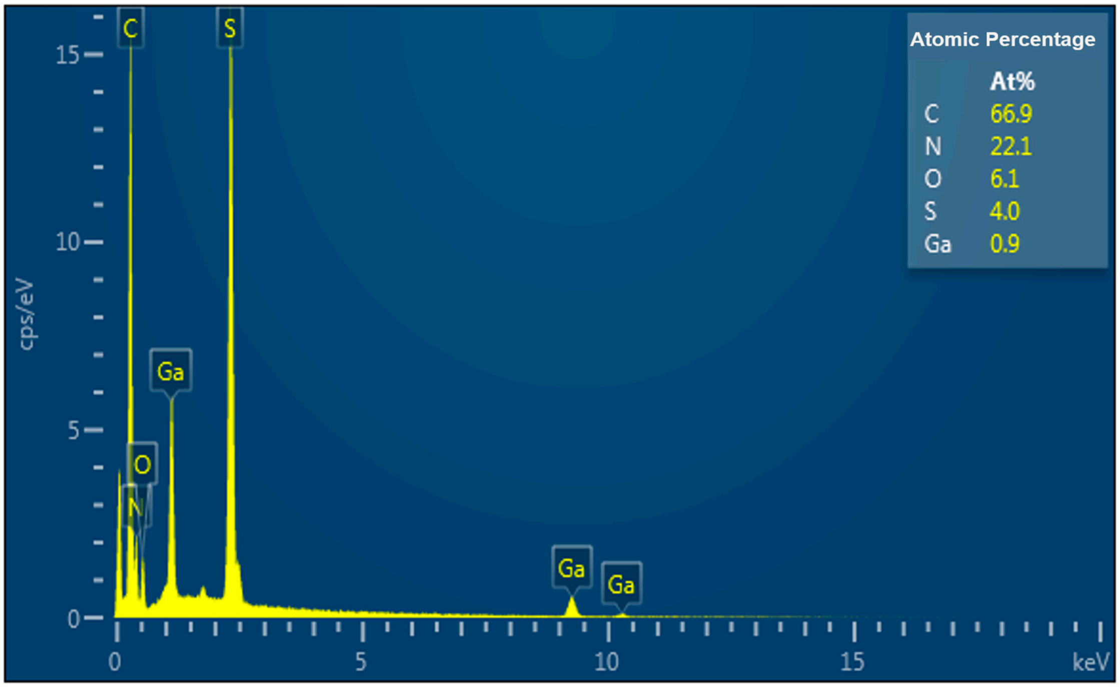

Figure S4 The EDS spectra of **[Ga(L)$_2$]NO$_3$**.

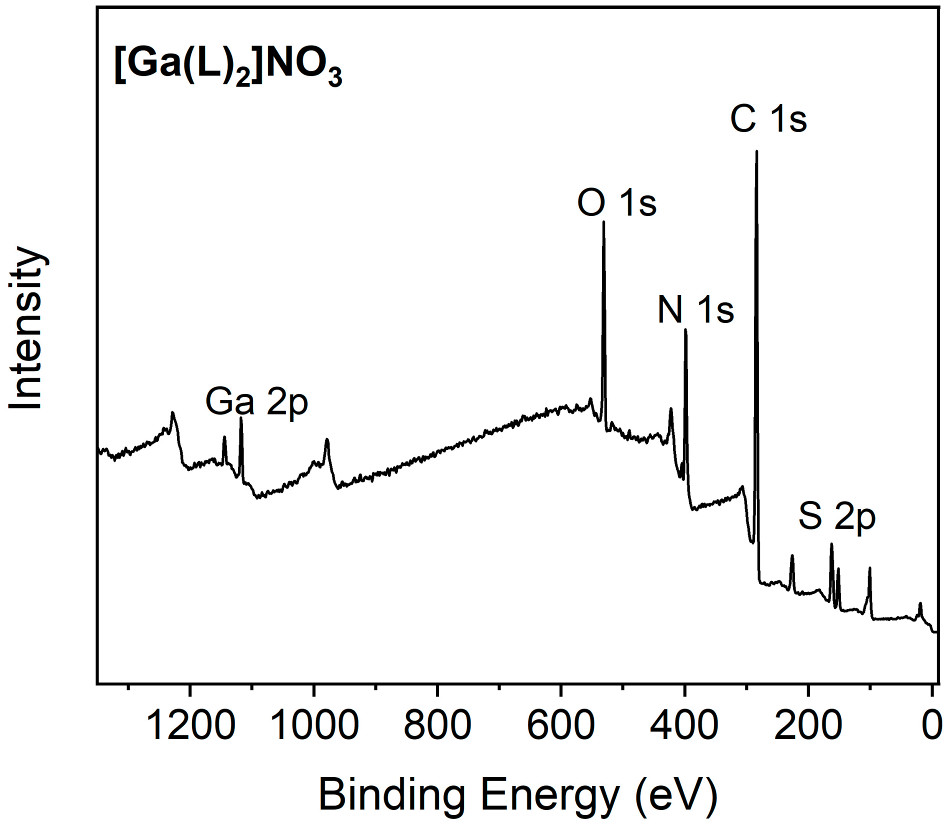

Figure S5 The full XPS spectra of **$[Ga(L)_2]NO_3$**.

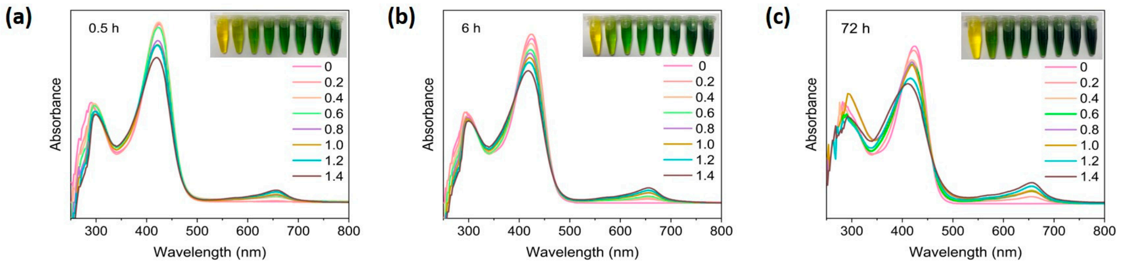

Figure S6 UV-Vis spectral and color (insets) changes of **[Ga(L)$_2$]NO$_3$** upon titration with Fe^{2+}, monitored at 0.5 h (a), 6 h (b), and 72 h (c).

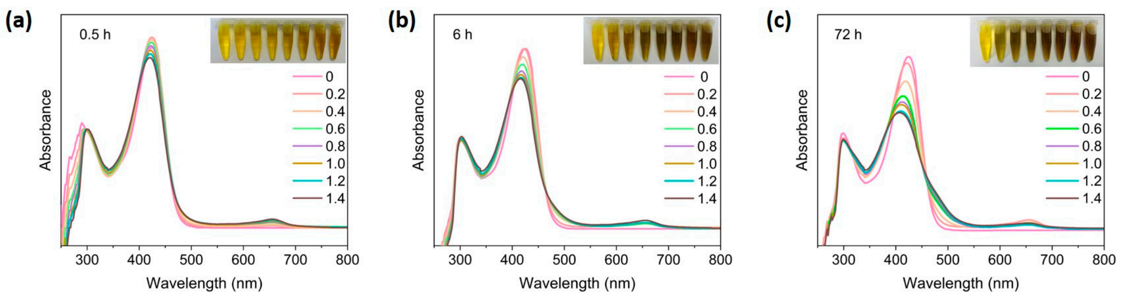

Figure S7 UV-Vis spectral and color (insets) changes of **[Ga(L)$_2$]NO$_3$** upon titration with Fe^{3+}, monitored at 0.5 h (a), 6 h (b), and 72 h (c).

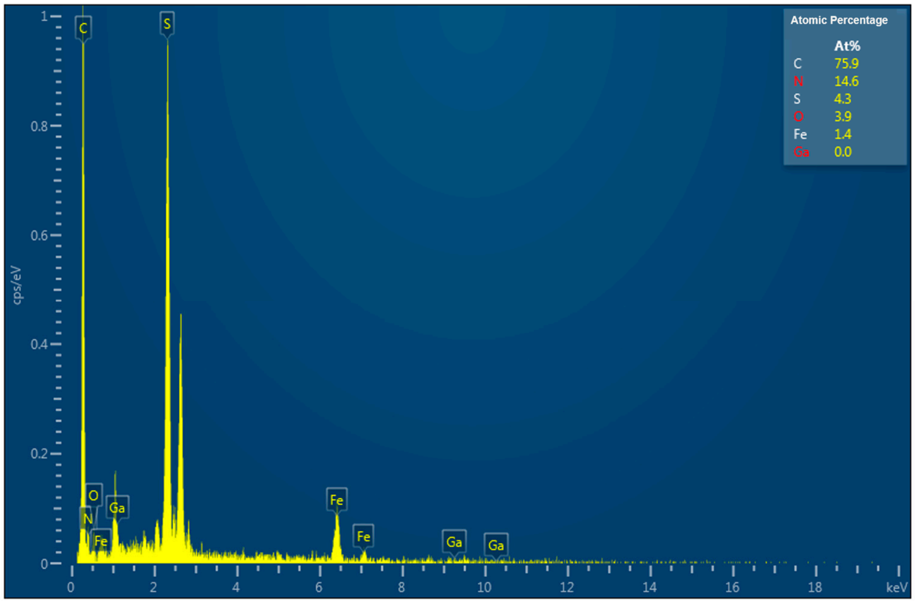

(**a**)

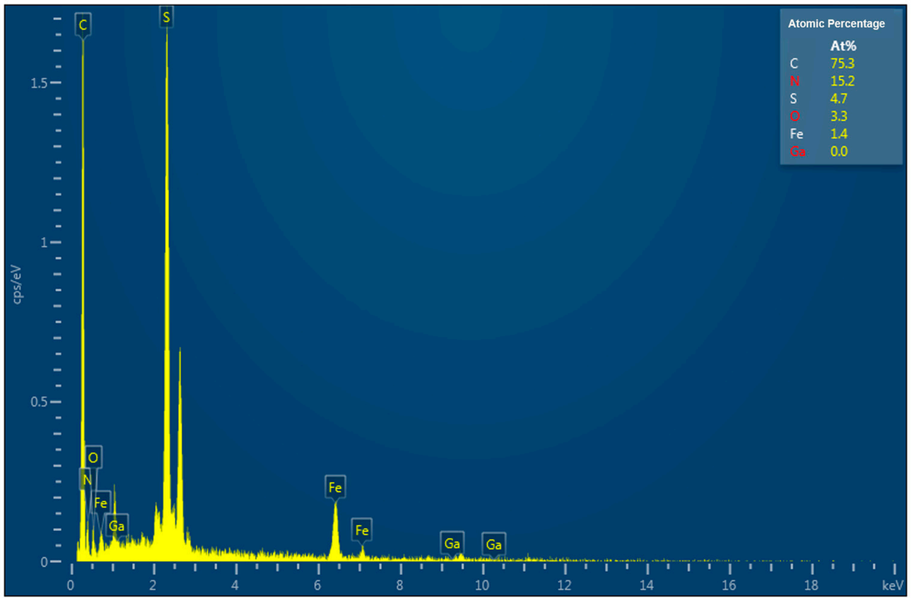

(**b**)

Figure S8 The EDS spectra of the solid products obtained from the reaction of **[Ga(L)$_2$]NO$_3$** with Fe^{2+} (a) and Fe^{3+} (b).

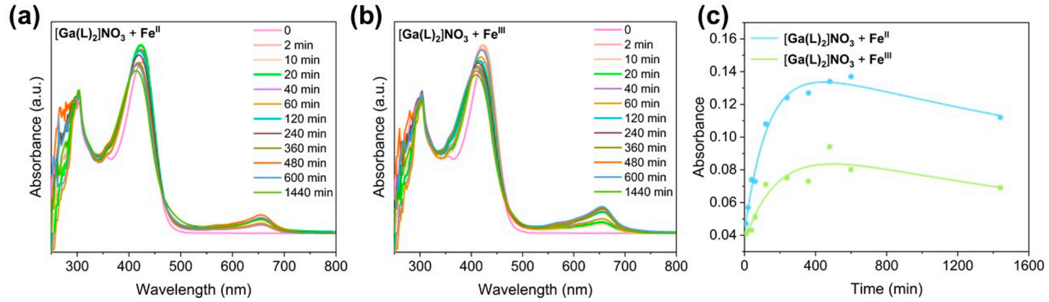

Figure S9 Time-resolved UV–Vis spectra of **$[Ga(L)_2]NO_3$** reacting with $(NH_4)_2Fe(SO_4)_2{\cdot}6H_2O$ (a) and $NH_4Fe(SO_4)_2{\cdot}12H_2O$ (b) at an molar ratio of 1 : 1 in DMF/H_2O at 60 °C (0–24 h). Theoretical fitting curves of the absorbance at 654 nm for (a) and (b) at different time intervals (c).

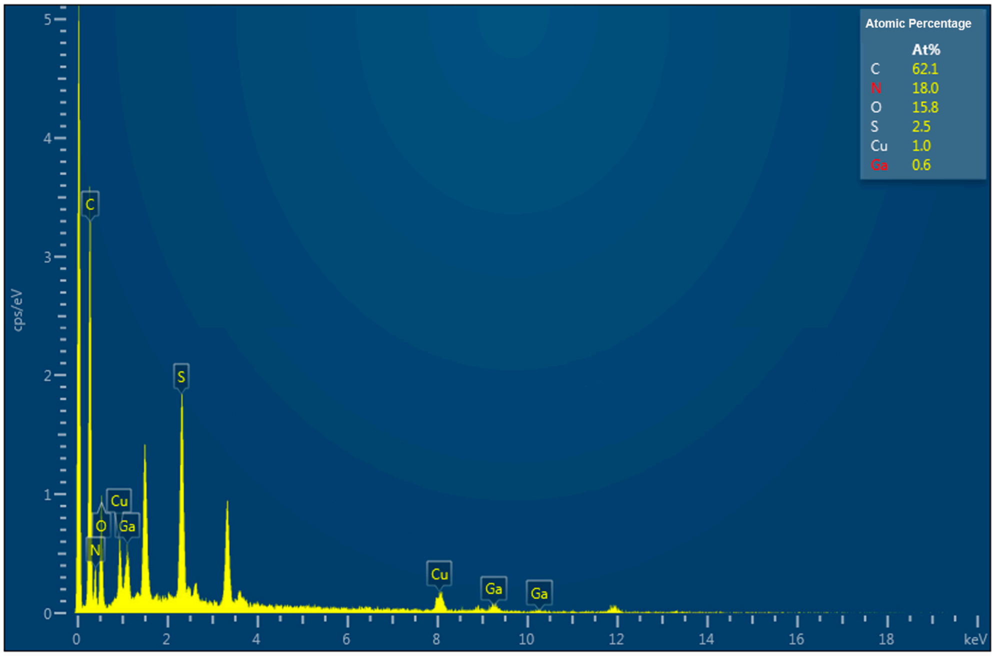
Atomic Percentage
At%
C 62.1
N 18.0
O 15.8
S 2.5
Cu 1.0
Ga 0.6
cps/eV
keV
C
N
O
Cu
Ga
S
Cu
Ga
Ga

(**a**)

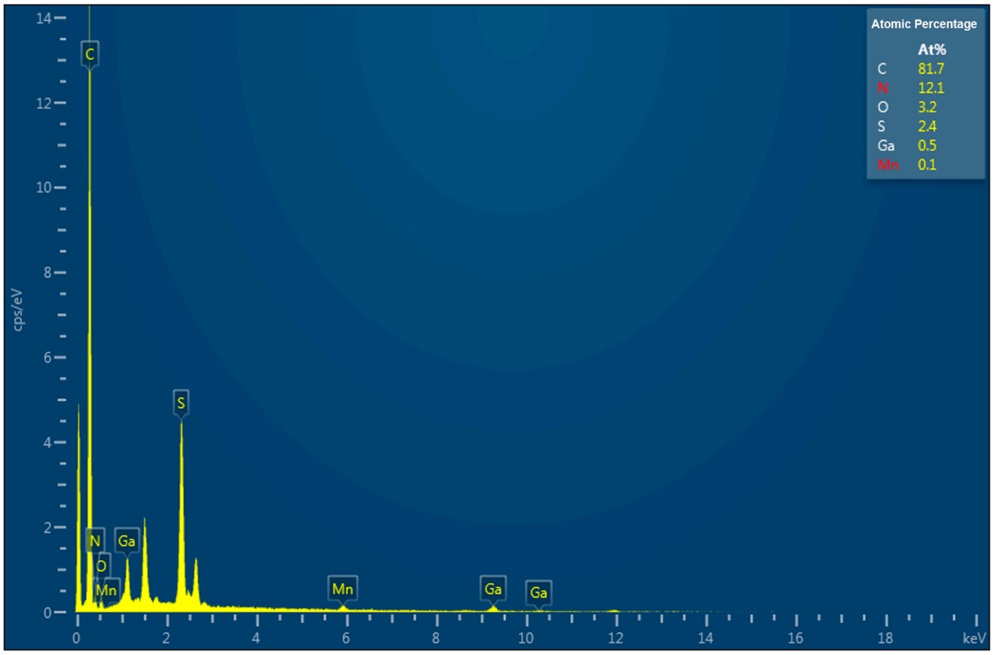
Atomic Percentage
At%
C 81.7
N 12.1
O 3.2
S 2.4
Ga 0.5
Mn 0.1
cps/eV
keV
C
N
O
Mn
Ga
S
Mn
Ga
Ga

(**b**)

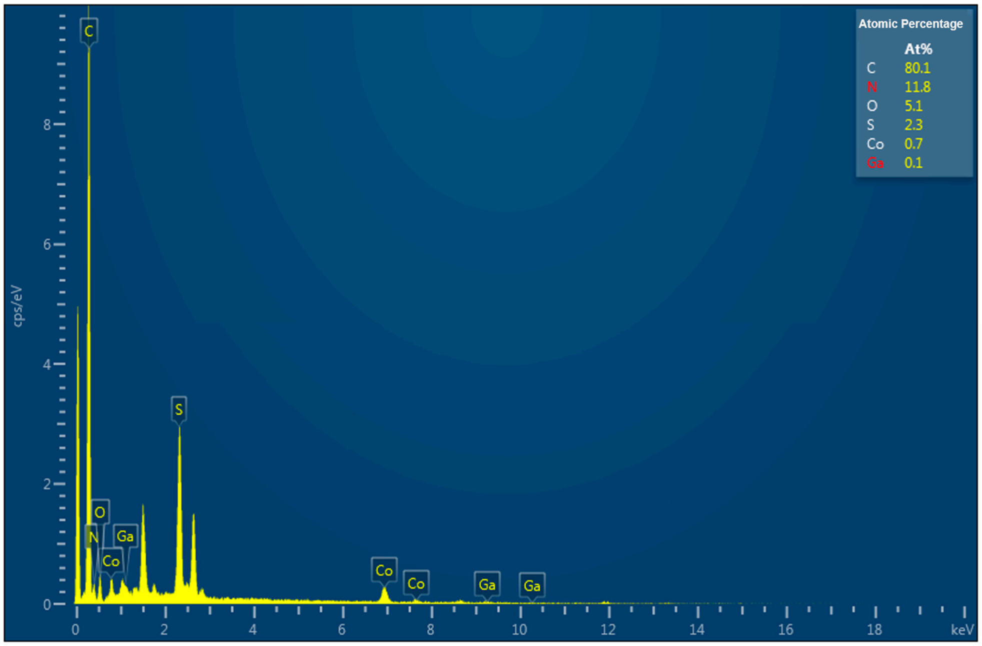
Atomic Percentage
At%
C 80.1
N 11.8
O 5.1
S 2.3
Co 0.7
Ga 0.1
cps/eV
keV

(c)

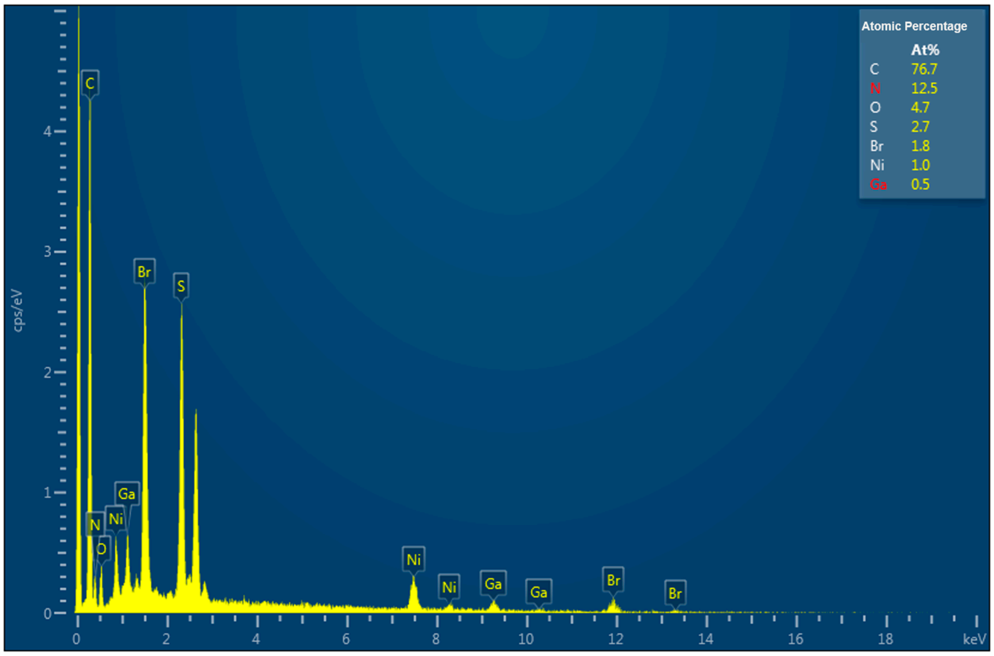
Atomic Percentage
At%
C 76.7
N 12.5
O 4.7
S 2.7
Br 1.8
Ni 1.0
Ga 0.5
cps/eV
keV

(d)

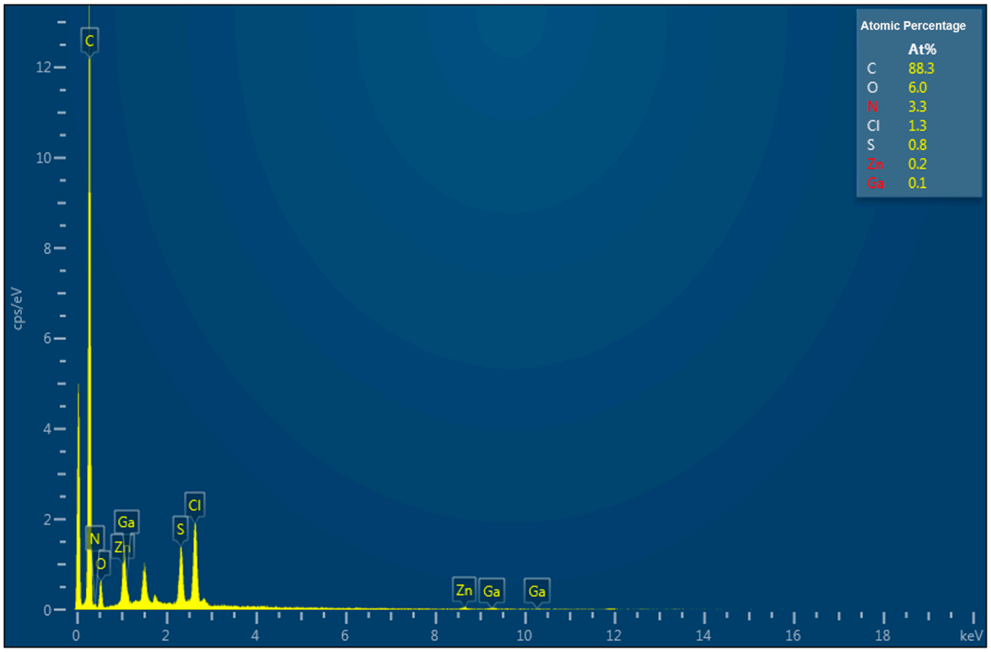

(**e**)

Figure S10 The EDS spectra of the solid products obtained from the reaction of **[Ga(L)$_2$]NO$_3$** with Cu(NO$_3$)$_2$·xH$_2$O (a), Mn(CH$_3$COO)$_2$·4H$_2$O (b), Co(CH$_3$COO)$_2$ (c), NiBr$_2$·xH$_2$O (d), and ZnCl$_2$ (e), showing the incorporation of different transition metal ions.

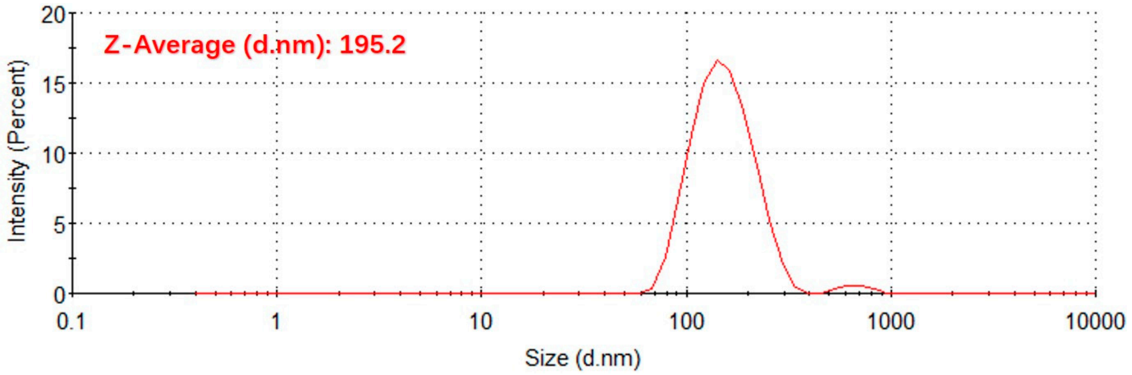

Figure S11 The DLS spectra of **[Ga(L)$_2$]A**.

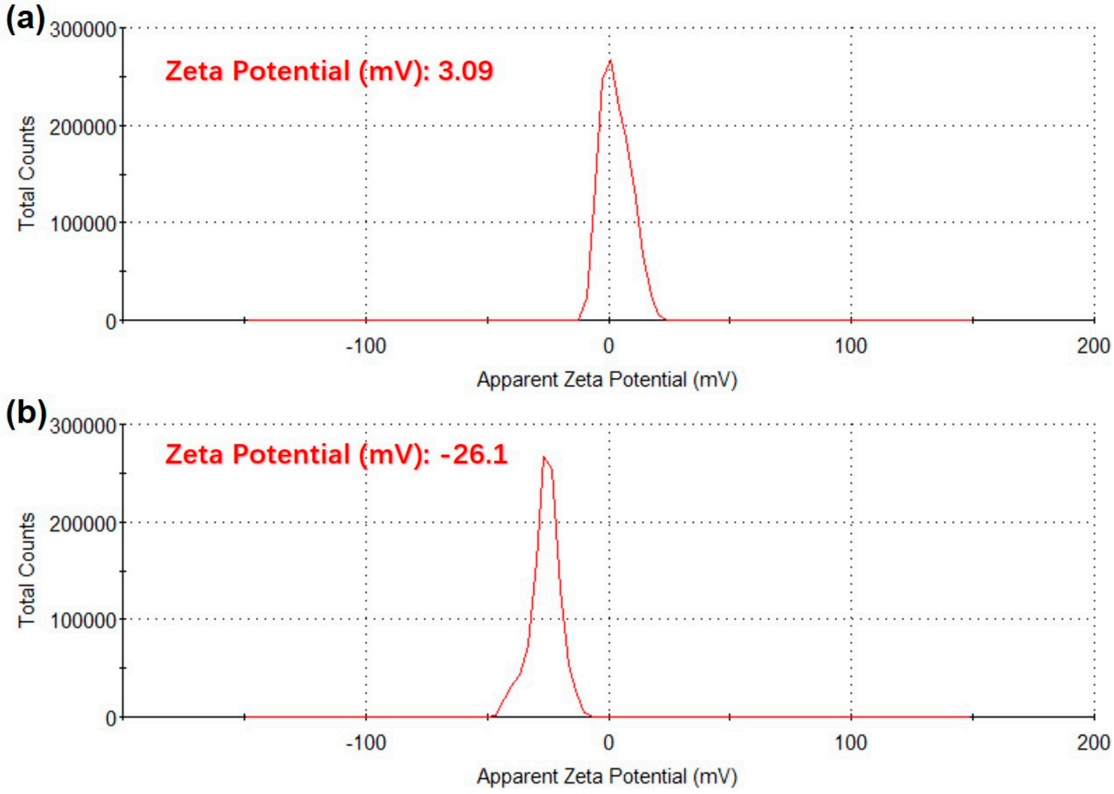

Figure S12 The zeta potential diagrams of **$[Ga(L)_2]NO_3$** (a) and **$[Ga(L)_2]A$** (b).

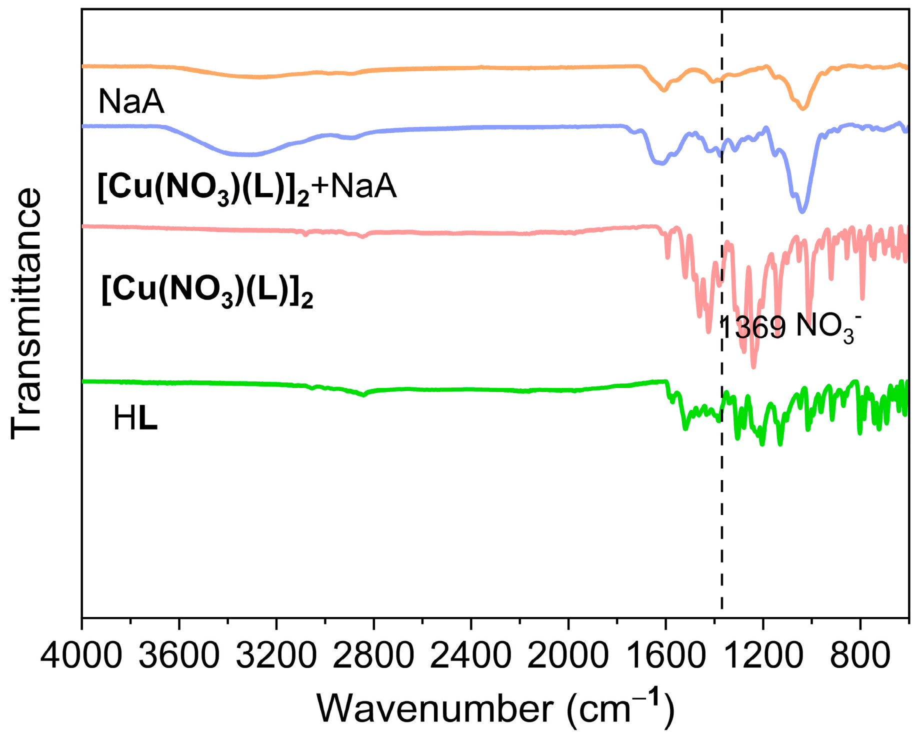

Figure S13. A comparison of the FT-IR spectra of H**L**, **[Cu(NO$_3$)(L)]$_2$**, **[Cu(NO$_3$)(L)]$_2$** + NaA, and NaA.

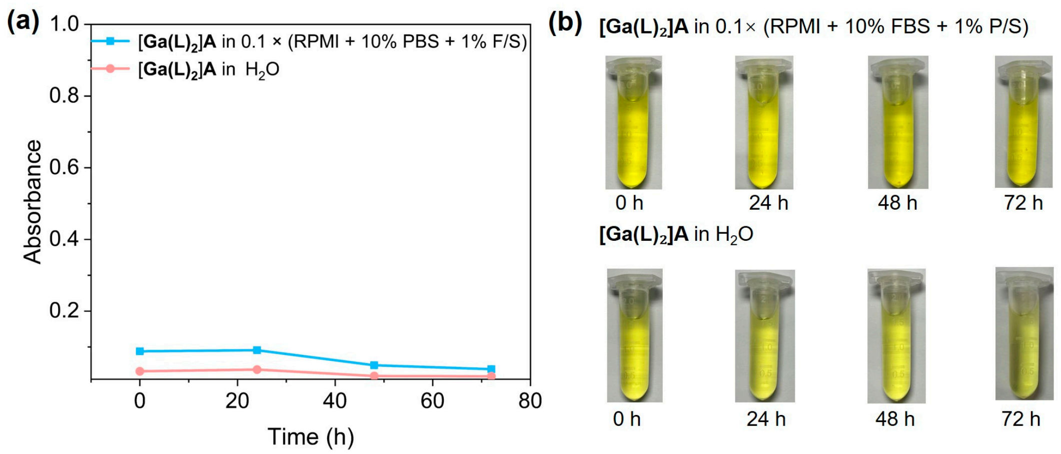

Figure S14 Turbidity-based stability assessment of **[Ga(L)₂]A** dispersions at the absorbance of 600 nm in 0.1 × (RPMI + 10% FBS + 1% P/S) and deionized water recorded at 0, 24, 48, and 72 h at room temperature (a). Photographs of **[Ga(L)₂]A** dispersions stored in 0.1 × (RPMI + 10% FBS + 1% P/S) and deionized water for 0, 24, 48, and 72 h at room temperature (b).

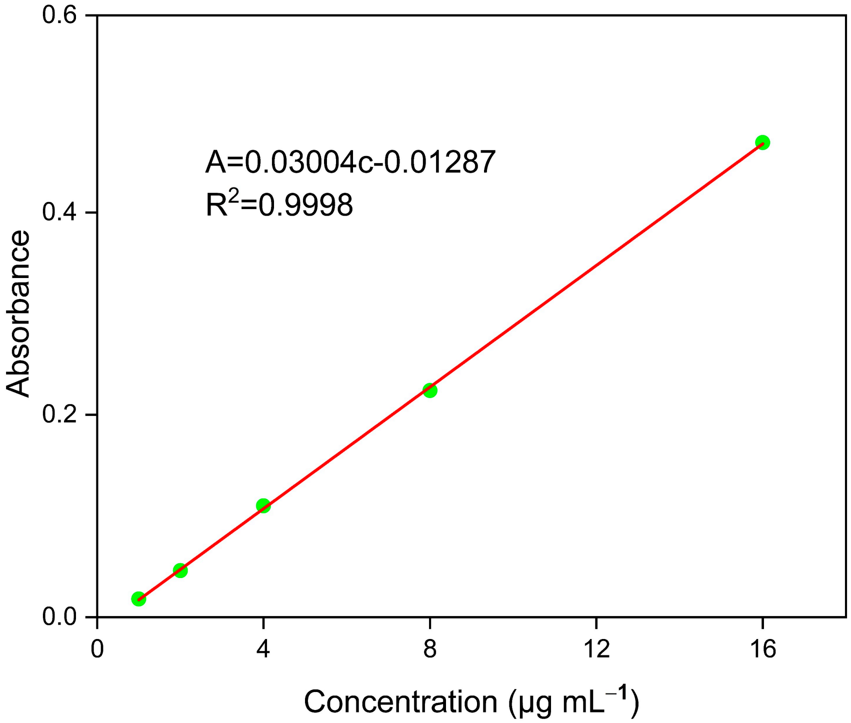

Figure S15 Standard curve of **$[Ga(L)_2]NO_3$** in deionized water. The plot depicts the absorbance at 420 nm as a function of concentration, showing a linear relationship in accordance with the Beer-Lambert law.

Table S1. Hydrogen bonding interactions for **[Ga(L)$_2$]NO$_3$** [Å and °].

D-H···A	d(D-H)	d(H···A)	d(D···A)	∠(DHA)
O(4)-H(4)···O(3)#1	0.83	1.92	2.750(4)	175.0
C(5)-H(5A)···O(3)#1	0.98	2.42	3.333(3)	154.3
C(7)-H(7A)···S(1)	0.98	2.49	3.023(2)	114.0
C(12)-H(12)···O(4)	0.94	2.61	3.515(4)	161.5
C(16)-H(16)···N(14)#2	0.94	2.60	3.405(3)	143.5
C(19)-H(19)···O(1)	0.94	2.55	3.254(3)	131.7
C(20)-H(20)···O(4)	0.94	2.52	3.363(4)	148.8
C(29)-H(29)···O(1)	0.94	2.56	3.418(4)	152.1
C(33)-H(33A)···O(2)	0.98	2.47	3.413(4)	161.8
C(35)-H(35A)···S(3)	0.98	2.48	3.020(3)	114.4
C(35)-H(35A)···N(1)#3	0.98	2.68	3.481(3)	139.0
C(35)-H(35B)···N(14)#4	0.98	2.53	3.442(3)	155.4

Symmetry transformations used to generate equivalent atoms:

#1 $-x + 3/2$, $y + 1/2$, $-z + 3/2$; #2 $-x + 3/2$, $y - 1/2$, $-z + 3/2$; #3 $-x + 2$, $-y + 1$, $-z + 1$; #4 $-x + 1$, $-y + 1$, $-z + 1$.
